# Comparison of transcatheter leaflet-approximation and direct annuloplasty in tricuspid regurgitation

**DOI:** 10.1007/s00392-023-02287-0

**Published:** 2023-08-29

**Authors:** Laurin Ochs, Maria Isabel Körber, Hendrik Wienemann, Tobias Tichelbäcker, Christos Iliadis, Clemens Metze, Monique Brüwer, Tobias Schmidt, Hazem Omran, Vera Fortmeier, Kai Friedrich, Volker Rudolph, Stephan Baldus, Roman Pfister

**Affiliations:** 1 Faculty of Medicine and University Hospital Cologne, Clinic III for Internal Medicine, University of Cologne, Kerpener Straße 62, 50937 Cologne, Germany; 2 Department of General and Interventional Cardiology, Heart and Diabetes Center Northrhine-Westfalia, Ruhr University Bochum, Bad Oeynhausen, Germany; 3 Medical Clinic II, University Heart Center Lübeck, University Hospital Schleswig-Holstein, Lübeck, Germany; 4 DZHK (German Center for Cardiovascular Research), Partner Site Hamburg/Kiel/Lübeck, Lübeck, Germany

**Keywords:** Tricuspid disease, Transcatheter tricuspid valve repair (TTVR), Access femoral, Leaflet-approximation, Annuloplasty, Cardioband

## Abstract

**Background:**

Transcatheter repair emerges as a treatment option in patients with tricuspid regurgitation (TR) and high surgical risk.

**Aims:**

This study aimed to compare leaflet-based and annuloplasty-based transcatheter repair in patients with TR.

**Methods:**

In a retrospective analysis consecutive patients undergoing either transcatheter edge-to-edge repair (TEER) or direct annuloplasty (AP) for relevant TR at 2 centers were compared with respect to baseline characteristics, procedural efficacy and safety (death, myocardial infarction, procedure or device-related cardiothoracic surgery, or stroke at 30 days).

**Results:**

161 patients (57% female, median age 79 [75–82] years) with comparable clinical baseline characteristics in the TEER (*n* = 87) and AP (*n* = 74) group were examined. Baseline TR grade was significantly less severe in the TEER compared to the AP group (torrential 9.2 vs. 31.1%, *p* = 0.001). Technical success and improvement of TR grades were not significantly different across groups. In analysis matched for baseline TR severity, reduction of TR grade to less than moderate was significantly more common in the AP group (47.8 vs. 26.1%, *p* = 0.031). Major or more severe bleeding occurred in 9.2% of TEER and 20.3% of AP patients (*p* = 0.049) without any fatal bleedings. Major adverse events (MAE) were similar across groups with four patients (4.7%) in the TEER group and five patients (6.9%) in the AP group (*p* = 0.733) and 6-month survival did not differ significantly.

**Conclusions:**

Differences observed between patients treated with TEER and AP provide first evidence for tailoring distinct transcatheter treatment techniques to individual patient characteristics.

**Graphic abstract:**

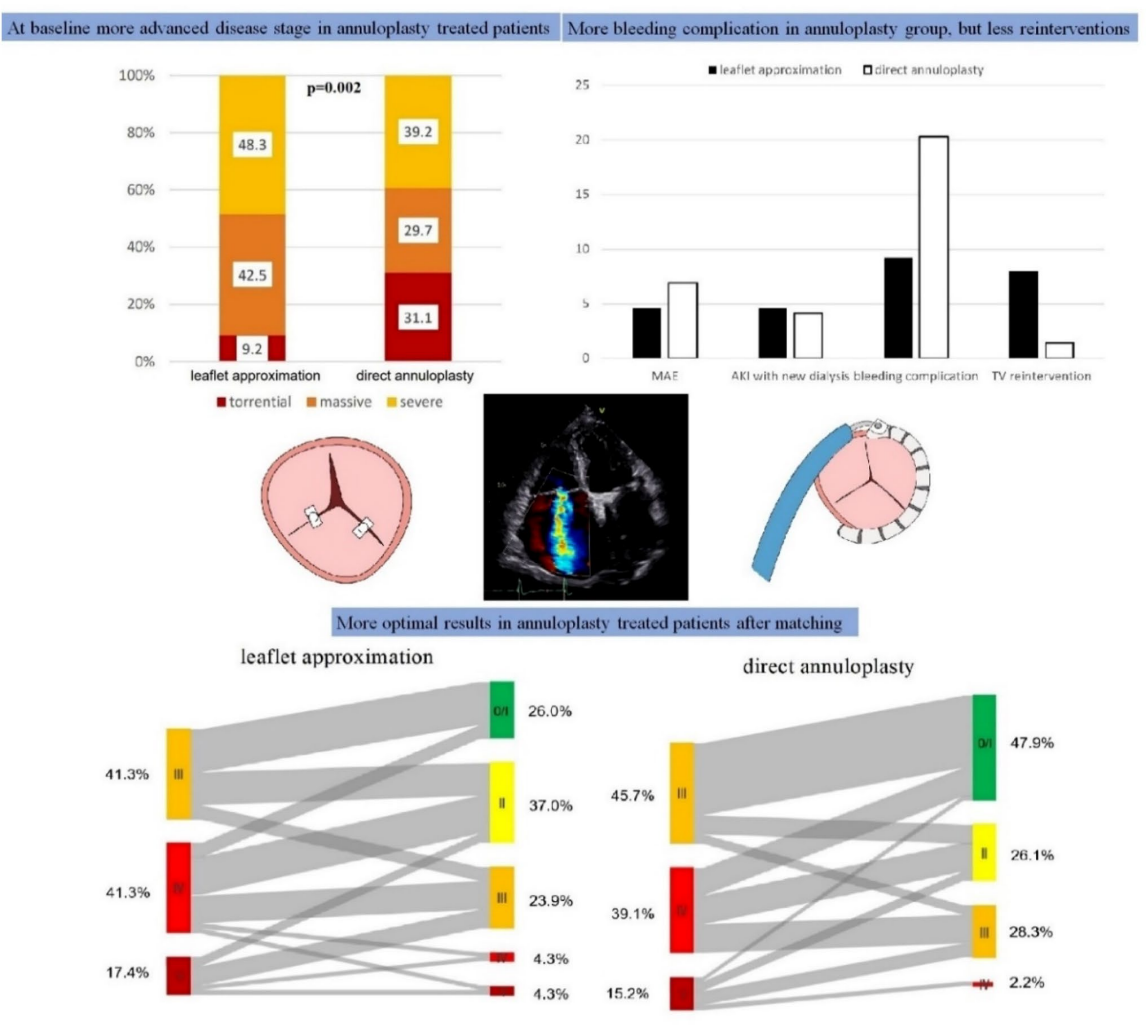

## Introduction

Clinically relevant tricuspid regurgitation (TR) is common in the community with a strong correlation with age and a prevalence of 3% in people aged 75 years or older [1]. More than 90% of TR cases are of secondary etiology and about 6% of patients with first diagnosis of heart failure either have or develop moderate-to-severe TR [2]. TR, even if isolated, is associated with excess mortality on the community level comprising an important public health problem [1, 3].

Until recently therapy options for the correction of TR were lacking. Only less than 3% of patients with moderate-to-severe TR undergo corrective surgery [1]. This is explained by the high surgical risk even in pre-selected patients [4]. Catheter-based therapies emerged as efficacious in improving TR with low periprocedural risk in preliminary observational studies [5–7]. In consequence, the 2021 Valvular Heart Disease guidelines of the European Society of Cardiology for the first time give a IIb level C recommendation for transcatheter treatment of severe symptomatic TR in inoperable patients [8].

The most commonly used transcatheter repair technique is transcatheter edge-to-edge repair (TEER). Different devices have been applied such as the MitraClip™ and lately the CE approved TriClip™ and PASCAL system [9–11]. The second transcatheter repair approach is based on narrowing the tricuspid annulus with direct annuloplasty (AP) using the CE approved Cardioband™ system [10, 12]. So far, comparative studies of both technical approaches with respect to efficacy and safety and subsequent recommendations on their differential use are lacking. Here, we analyzed consecutive patients treated with either of the above transcatheter repair techniques at two high volume centers and compared baseline characteristics and measures of procedural efficacy and safety.

## Methods

### Study population

In this bi-center retrospective analysis we included all consecutive patients of our prospective database of patients treated for tricuspid regurgitation with TEER or AP between 2017 and 2020 at the Heart Centre of the University Hospital of Cologne and between 2017 and 06/2021 at the Heart Centre Bad Oeynhausen. All patients had symptomatic tricuspid regurgitation of severe grade or more and were considered as high operative risk by the heart team with a decision for a catheter-based approach. Feasibility of the two repair techniques was evaluated by local imaging specialists and interventional cardiologists performing both techniques, and anatomical suitability of annuloplasty was additionally evaluated by the manufacturer based on cardiac computed tomography. AP was performed with the Cardioband^®^ (Edwards Lifesciences, Irvine, California) and TEER with the TriClip^®^, MitraClip^®^ (both Abbott Vascular, Chicago, IL, USA) or PASCAL^®^ system (Edwards Lifesciences, Irvine, CA, USA), as available and decided by the treating interventional cardiologist. During the study time, TEER was the standard technique and first choice for transcatheter valve repair in both centers. Criteria for AP treatment were anatomic or structural conditions potentially impairing leaflet grasping, i.e., primarily a large coaptation gap, but also short or restrictive leaflets, leaflet indentations or pacemaker leads in the region of the intended grasping. A total of 134 patients were evaluated for AP during the study period of which 76 patients were finally treated (Supplemental Fig. 1). Two patients of this group were excluded from AP group due to previous TEER. 14 patients were canceled due to patient reasons (denial or relevant clinical deterioration), 44 patients were rejected due to RCA proximity and annulus size. 17 of the latter were treated conservatively, 10 patients underwent high risk tricuspid surgery and 17 were treated with TEER with inclusion for analysis in TEER group. 57 patients from TEER group and 35 from AP group were treated at the Heart Centre Bad Oeynhausen, while 30 patients from TEER group and 39 from AP group underwent treatment at the Heart Centre of the University Hospital of Cologne.

Transcatheter valve replacement and other transcatheter repair techniques were not available at both centers during the study time. Patients with concomitant mitral valve intervention and patients with a prior percutaneous treatment of the tricuspid valve were excluded.

This study was conducted in accordance with the declaration of Helsinki. Written informed consent was collected from all patients prior to the intervention for participation in a prospective local registry and data were retrieved from digital patient files. Follow-up data were obtained either from follow-up visits or via contacting general practitioner or the patient.

### Echocardiographic evaluation and procedure

TR grade and morphology were determined by echocardiographic evaluation of valvular heart disease before procedure and postprocedural in accordance with current guidelines [13, 14]. TR was graduated retrospectively into five grades according to classification of Hahn et al. as following: none (0), mild (I), moderate (II), severe (III), massive (IV) and torrential (V) [15].

All procedures were performed in general anaesthesia and transesophageal echocardiographic and fluoroscopic guidance as reported previously [12, 16]. During every Cardioband procedure coronary angiography was performed and a guide wire was inserted as a landmark for the implantation and to assess potential right coronary artery (RCA) damage.

### Outcomes

Outcomes were assessed according to Mitral Valve Academic Research Consortium (MVARC) unless indicated otherwise [17]. Efficacy endpoints according to MVARC were: (1) acceptable reduction of TR defined by improvement of at least one grade and to moderate or less and (2) optimal reduction of TR defined by improvement of at least one grade and to less than moderate TR. Further efficacy outcomes analyzed were reduction of at least one, two and three grades from baseline.

The primary safety endpoint was the composite of individual major adverse events (MAE) defined in accordance with MVARC criteria: periprocedural myocardial infarction, need for urgent cardiothoracic surgery and stroke or death within postprocedural 30 days or intrahospital. Furthermore, we assessed bleeding and acute kidney injury including necessity of dialysis in postprocedural 30 days, both according to MVARC criteria, and bleeding necessitating intervention and bleeding associated with red blood cell transfusion.

### Statistical analysis

Baseline characteristics and outcomes were compared by treatment groups (TEER vs. AP). Normal distribution of variables was examined with the Kolmogorov–Smirnov test. For comparison of metric variables Mann–Whitney *U* test or *t* test, and for ordinary variables Chi-square and exact-Fisher test were used, as appropriate. For paired data Wilcoxon signed rank test was applied. Since treatment groups differed significantly by baseline characteristics which potentially impact treatment efficacy, in secondary analysis groups were matched according to these covariates: tricuspid regurgitation grade at baseline, presence of trans-tricuspid pacemaker lead, vena contracta, coaptation gap and age. The survival analysis was performed using Kaplan Meier analysis and plots were truncated at 6 months. A two-tailed *p* value < 0.05 was regarded as statistically significant. Statistical analysis was performed with IBM SPSS Statistics version 28.0 (Chicago, IL). Matching was performed with R version 4.1 (Vienna, Australia) and R Studio by Full Matching method using MatchIt package.

## Results

### Patient characteristics of total cohort

We enrolled a total of 161 patients of whom 87 underwent TEER and 74 underwent AP. In the TEER group 30 patients underwent PASCAL implantation, 37 patients underwent MitraClip implantation in tricuspid position and 15 patients underwent TriClip implantation.

Baseline characteristics are summarized in Table 1. Patients of both groups were at high operative risk due to age (80 [76–83] vs. 78 [73–82]), comorbidities and with a median intermediate risk EuroScore II of 5.9 [3.3–11.1, mean 7.8%] % and 4.5 [2.5–7.4, mean 6.2%] %. Patients were highly symptomatic with mostly NYHA class III/IV (97.7 vs. 95.9%). Groups differed significantly with respect to higher age, more men, more patients with transvalvular pacemaker leads and coronary heart disease and less patients with diabetes mellitus in the TEER group (Table 1).

**Table 1:** Patient characteristics of the total cohort and by treatment technique

	All *n* = 161	TEER group *n* = 87	AP group *n* = 74	*p* value
Age, years	79 [75–82]	80 [76–83]	78 [73–82]	0.031
Female	92 (57.1)	39 (44.8)	53 (71.6)	< 0.001
Body mass index, kg/m^2^	25.5 [22.4–29.0]	24.8 [22.0–28.7]	26.1 [22.5–29.6]	0.202
EuroScore II (median), %	5.3 [3.0–8.5]	5.9 [3.3–11.1]	4.5 [2.5–7.4]	0.057
EuroScore II (mean), %	7.1 ± 7.7	7.8 ± 8.2	5.9 ± 6.8	0.168
NYHA functional class				0.180
II	5 (3.1)	2 (2.3)	3 (4.1)	
III	140 (87.0)	73 (83.9)	67 (90.5)	
IV	16 (9.9)	12 (13.8)	4 (5.4)	
Coronary artery disease	74 (46.0)	49 (56.3)	25 (33.8)	0.004
Diabetes mellitus	38 (23.6)	14 (16.1)	24 (32.4)	0.015
Prior stroke	32 (19.9)	16 (18.4)	16 (21.6)	0.609
COPD	26 (16.1)	10 (13.0)	16 (21.6)	0.082
Prior dialysis	9 (5.6)	3 (3.4)	6 (8.1)	0.303
Prior open heart surgery	59 (36.6)	32 (36.8)	27 (36.5)	0.969
Prior left-sided transcatheter valve replacement or repair	11 (6.8)	4 (4.6)	7 (9.5)	0.223
Atrial fibrillation	139 (86.3)	73 (83.9)	66 (89.2)	0.660
Pacemaker lead in RV	39 (24.2)	29 (33.3)	10 (13.5)	0.003
Estimated glomerular filtration rate (CKD EPI), ml/min/m^2^	46 [31–61]	43 [28–58]	48 [33.75–65]	0.125
GOT, IU/l	30.5 [25.75–38.0]	31.0 [25.0–38.75]	30.0 [26.0–38.0]	0.978
GPT, IU/l	18.0 [14–25.5]	19.5 [15.0–27.75]	17.0 [13.0–22.0]	0.048
Hemoglobin, g/dl	11.6 ± 2.1	11.7 ± 2.0	11.6 ± 2.1	0.666
NT-pro-BNP, pg/ml	2.374 [1.252–4.594]	2.490 [1.250–6262]	1.970 [1.256–3998]	0.132
Prior MVR	10 (6.2)	3 (3.4)	7 (9.5)	0.115
Prior PMVR	18 (11.2)	9 (10.3)	9 (12.2)	0.715
LV-EF (mean), %	52.2 ± 10.7	49.6 ± 11.8	55.3 ± 8.0	< 0.001
Patients with reduced LV-EF	22 (13.7)	34 (39.1)	22 (29.7)	0.290
Grade of tricuspid regurgitation				0.002
Severe	71 (44.1)	42 (48.3)	29 (39.2)	
Massive	59 (36.6)	37 (42.5)	22 (29.7)	
Torrential	31 (19.3)	8 (9.2)	23 (31.1)	
TR-EROA, mm^2^	30.0 [0.9–60.0]	30.0 [0.7–50.0]	25.0 [1.0–70.0]	0.400
Basal RV diameter, mm	48.2 ± 7.8	48.0 ± 8.2	48.5 ± 7.4	0.664
TAPSE, mm	16.7 ± 4.5	16.1 ± 4.7	17.5 ± 4.2	0.046
FAC, %	39.1 ± 12.0	38.3 ± 12.1	40.1 ± 11.9	0.369
Tricuspid annulus diameter (anteroseptal), mm	43.1 ± 6.1	43.1 ± 5.6	43.1 ± 6.5	0.955
Vena contracta, mm	11.0 [8.0–14.8]	10.0 [8.0–14.0]	13.0 [8.0–16.0]	0.022
Coaptation gap, mm	4.3 [0.0–7.3]	2.0 [0.0–6.3]	5.0 [3.0–11.25]	< 0.001
PAmean in RHC	28.5 ± 8.1 (*n* = 140)	29.0 ± 9.0 (*n* = 75)	27.9 ± 7.0 (*n* = 65)	0.428
PCWP in RHC	19.3 ± 7.4 (*n* = 138)	19.5 ± 7.7 (*n* = 73)	19.0 ± 7.1 (*n* = 65)	0.702

*COPD* chronic obstructive pulmonary disease, *FAC* fractional area change, *LV-EF* left ventricular ejection fraction, *MVR* mitral valve repair/replacement, *NT-proBNP* N-terminal pro-brain natriuretic peptide, *NYHA* New York Heart Association, *PAmean* mean pulmonary arterial pressure, *PCWP* pulmonary capillary wedge pressure, *PMVR* percutaneous mitral valve repair, *RHC* right heart catheterization, *RV* right ventricle, *TR-EROA* tricuspid regurgitation effective regurgitant orifice area, *TAPSE* tricuspid annular plane systolic elevation

**Fig. 1:**
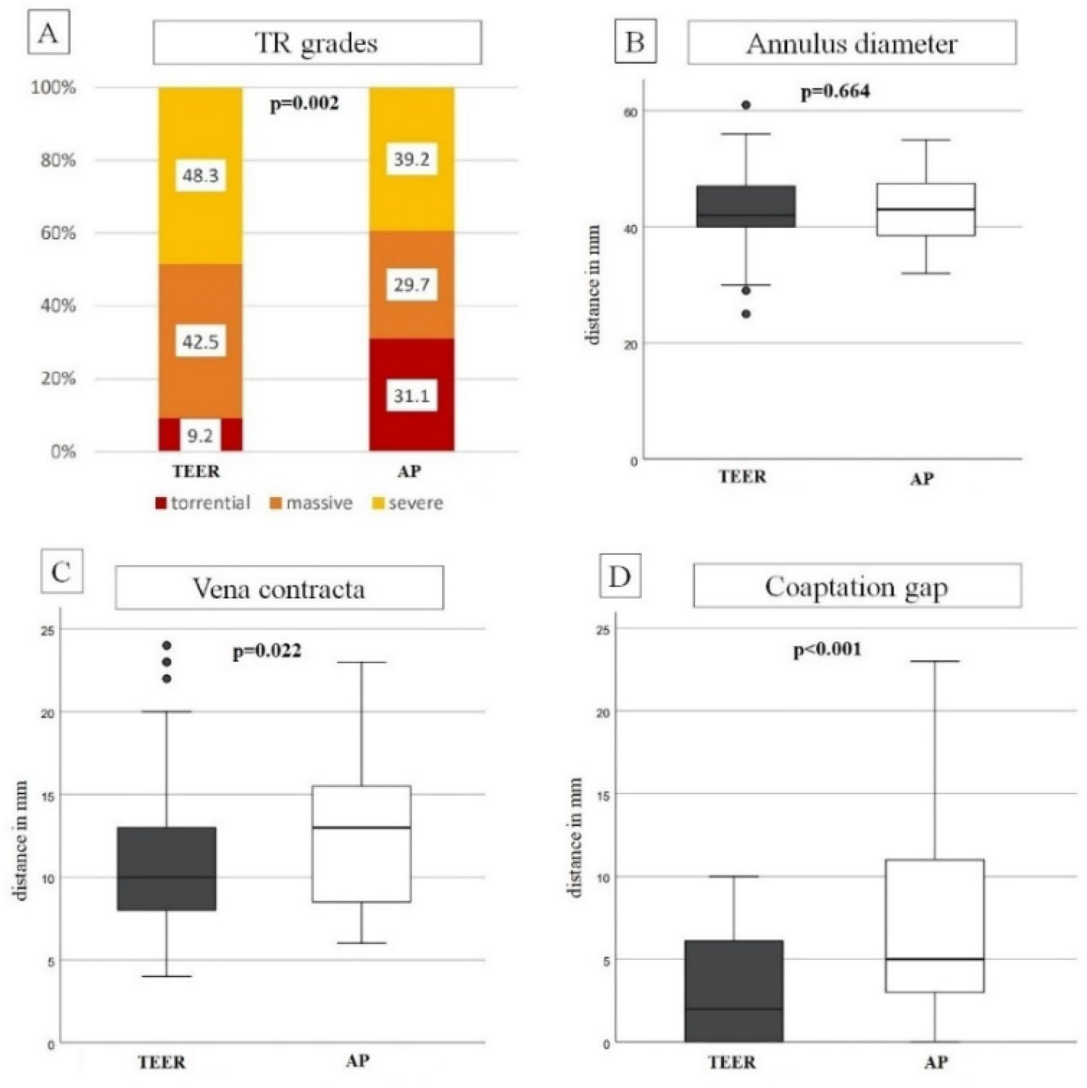
Preprocedural echocardiographic parameters. Comparison of preprocedural grade of TR, anteroseptal annulus diameter, coaptation gap and vena contracta measured by echocardiography. More patients in the annuloplasty group showed torrential TR (**A**). While annulus diameter was comparable in both groups (**B**), coaptation gap and vena contracta were significantly larger in the annuloplasty group (**C**, **D**). *TR* tricuspid regurgitation, *TEER* transcatheter edge to edge repair, *AP* direct annuloplasty

Baseline tricuspid regurgitation grade differed significantly between groups with less torrential cases in the TEER group (Fig. 1). Accordingly, in the AP group mean coaptation gap and vena contracta were significantly larger. No significant differences were observed for RV basal diameter and anteroseptal tricuspid annulus diameter. Right ventricular function evaluated by tricuspid annular plane systolic excursion (TAPSE) was slightly worse in the TEER group which was of borderline significance and fractional area change (FAC) showed no significant differences between groups (38.3 vs. 40.1%, *p* = 0.368).

### Procedural outcomes of total cohort

Procedural characteristics are presented in Table 2. Procedural time was significantly longer in the AP group than in the TEER group with a difference in median time of 85 min (*p* < 0.001). The length of hospital stay was significantly longer in the AP group with a difference in median length of 0.5 days (*p* < 0.001).

**Table 2:** Procedural details of the total cohort and by treatment technique

	All *n* = 161	TEER group *n* = 87	AP group *n* = 74	*p* value
Length of stay in hospital, days	7 [5–10]	6 [4–10]	8 [7–11]	< 0.001
Procedure time, minutes	167 [122–212]	124 [83–172]	209 [170–243]	< 0.001
Contrast-medium volume, ml			94 [73–148]	
Device details				
1/2/3 Pascal devices (*n* = 30)		7/20/3		
1/2/3 MitraClips (*n* = 37)		11/22/4		
1/2/3 TriClips (*n* = 15)		4/8/3		
0/1/2/3 devices (total)		5/22/50/10		
Cardioband size C/D/E/F			2/3/19/50	
Technical success	152 (94.4)	80 (92.0)	72(97.3)	0.498
TR reduction of ≥ 1 grades	139 (86.3)	75 (86.2)	64 (86.5)	0.930
TR reduction of ≥ 2 grades	100 (62.1)	51 (58.6)	49 (66.2)	0.309
TR reduction of ≥ 3 grades	40 (24.8)	16 (18.4)	24 (32.4)	0.039
TR reduction to grade ≤ 2	109 (67.7)	60 (69.0)	49 (66.2)	0.720
TR reduction to grade ≤ 1	56 (34.8)	29 (33.3)	27 (36.5)	0.740
Trans-tricuspid gradient, mmHg	2.0 ± 1.1	2.4 ± 1.2	1.5 ± 0.7	< 0.001

*TR* tricuspid regurgitation

**Fig. 2:**
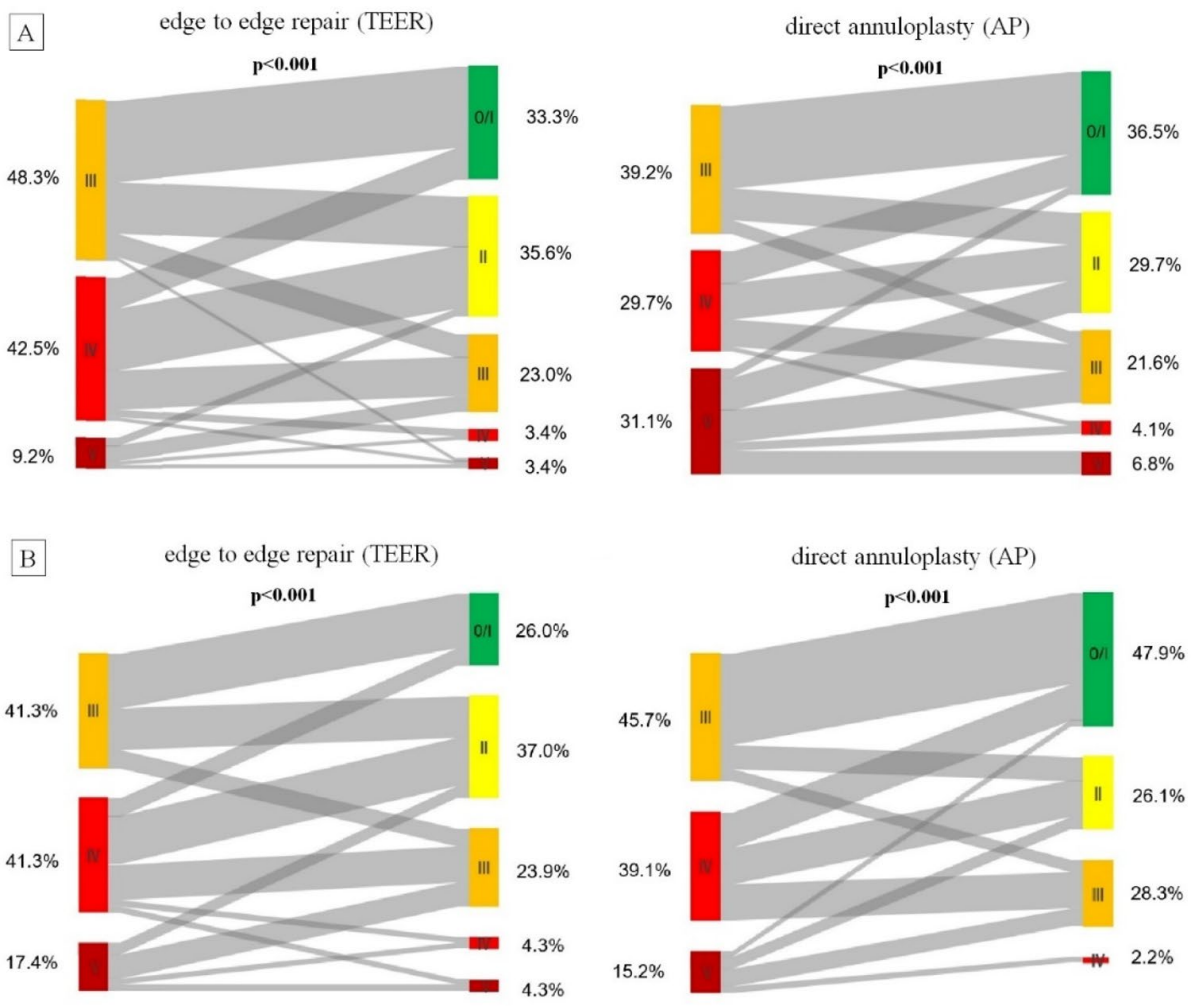
Comparison of TR reduction in total (**A**) and matched (**B**) cohort. Changes of TR from preprocedural to postprocedural comparing transcatheter edge to edge repair and direct annuloplasty is shown for total cohort (**A**) and the propensity-matched cohort (**B**). TR improved significantly in both groups (*p* < 0.001). While initially there were more advanced TR stages in annuloplasty group after intervention TR grades were similar (**A**). After matching initially TR grades were comparable and in the annuloplasty group an optimal reduction of TR grade to mild or none was significantly more common (26.1 vs. 47.8%, *p* = 0.031). (0 = none, I = mild, II = moderate, III = severe, IV = massive, V = torrential, TR = tricuspid regurgitation)

Technical success was 92.0% in the TEER group and 97.3% in the AP group (*p* = 0.498). In 5 patients of the TEER group a device placement was not successful (PASCAL device was intended in 2 patients and Mitra-Clip in 3 patients). TR grade improved significantly in both groups with a reduction of at least one grade in 86.2% of the TEER group and in 86.5% of the AP group (*p* = 0.930, Fig. 2A). An optimal reduction to TR grade mild or less was achieved similarly in both groups in about one-third of the patients (33.3 vs. 36.5%, *p* = 0.740) and an acceptable reduction of postprocedural TR to moderate or less was achieved in about two-third of patients (69 vs. 66.2%, *p* = 0.720), respectively. Significantly more patients in the AP group had an improvement in TR of three grades or more (18.4 vs. 32.4%, *p* = 0.039). The postprocedural trans-tricuspid gradient was significantly higher in the TEER compared to the AP group (2.4 vs. 1.5 mmHg, *p* < 0.001).

### Procedural outcomes of matched cohort

The matched cohort comprised 46 patients in each treatment group and was well-balanced for covariables relevant for the procedural success (Supplemental Table 1). There was no evidence for gender differences in the outcomes of Table 2, so we only show results of the gender combined cohort. The technical success (91.3 vs. 97.8%, *p* = 0.361) and reduction of at least one TR grade (87.0 vs. 95.7%, *p* = 0.139) did not differ significantly between groups (Supplemental Table 2). Figure 2B shows the changes of TR grade for the matched groups. Significantly more patients in the AP group achieved an optimal reduction of TR grade to mild or none (26.1 vs. 47.8%, *p* = 0.031), while the rate of an acceptable result with TR reduction to moderate or less was similar across groups (*p* = 0.262).

### Safety outcome of total cohort

The 30-day outcomes of total cohort are presented in Table 3. One patient of each group was lost to follow-up after transfer to another hospital on days 6 and 9, respectively. MAEs were similar across groups with four patients (4.7%) in the TEER group and five patients (6.9%) in the AP group (*p* = 0.733). Eight of the nine events were deaths. In the TEER group deaths occurred at days 8, 10, 49 and 140, with the latter two after prolonged intra-hospital course after secondary surgical tricuspid valve replacement and one due to septic shock after wound infection. In the AP group deaths occurred at days 3, 5, 13, and 29 with two fatal arrythmias on days 3 and 5 after intervention and one stroke leading to aspiration pneumonia and death on day 29. One patient developed a type two myocardial infarction due to anemia and with exclusion of relevant coronary stenosis with subsequent arrythmia in context of impaired left-ventricular function leading to death. The ninth event was a patient who underwent immediate surgery due to pericardial tamponade after RCA perforation and who recovered while intra-hospital course.

**Table 3:** Postprocedural outcomes of the total cohort and by treatment technique

	All *n* = 161	TEER group *n* = 87	AP group *n* = 74	*p* value
Major adverse events^a^	9 (5.6)	4 (4.6)	5 (6.9)	0.733
Death within 30 days	6 (3.7)	2 (2.3)	4 (5.4)	0.413
Death intrahospital	8 (5.0)	4 (4.6)	4 (5.4)	1.000
Myocardial infarction	1 (0.6)	0 (0.0)	1 (1.4)	0.456
Stroke	1 (0.6)	0 (0.0)	1 (1.4)	0.452
Procedure or device-related open heart surgery	3 (1.9)	2 (2.3)	1 (1.4)	1.000
Renal failure	9 (5.6)	2 (2.3)	7 (9.5)	0.080
Safety endpoint according to TRILUMINATE^b^	17 (10.6)	7 (8.0)	10 (13.5)	0.261
Acute renal failure with new dialysis	7 (4.3)	4 (4.6)	3 (4.1)	1.000
Access-site complication	6 (3.7)	1 (1.1)	5 (6.8)	0.061
Bleeding complications (MVARC overall)	23 (14.3)	8 (9.2)	15 (20.3)	0.049
Major bleeding	11 (6.8)	3 (3.4)	8 (10.8)	
Extensive bleeding	6 (3.7)	1 (1.1)	5 (6.8)	
Life-threatening bleeding	6 (3.7)	4 (4.6)	2 (2.7)	
Fatal bleeding	0	0	0	
Bleeding requiring intervention	18 (11.2)	3 (3.4)	15 (20.3)	< 0.001
Red blood cell transfusion	19 (11.8)	4 (4.6)	15 (20.3)	0.002
Reintervention at tricuspid valve	8 (5.0)	7 (8.0)	1 (1.4)	0.061
NYHA functional class at follow-up^c^				0.001
I	10 (8.0)	4 (6.0)	6 (10.3)	
II	59 (47.2)	21 (31.3)	38 (65.5)	
III	53 (42.4)	39 (58.2)	14 (24.1)	
IV	3 (2.4)	3 (4.5)	0 (0.0)	
Improvement ≥ 1 NYHA classes	74 (46.0)	31 (35.6)	43 (58.1)	0.019

*MVARC* Mitral Valve Academic Research Consortium, *NYHA* New York Heart Association

^a^Multiple major adverse event in one patient possible

^b^cardiovascular mortality, myocardial infarction, stroke, renal failure, non-elective surgery[28]

^c^22 patients had no NYHA class available at follow-up and 6 patients were excluded, since device was not implanted as intended

Major or more severe bleeding events according to MVARC were significantly more common in the AP group (20.3 vs. 9.2%). No difference was observed for life-threatening bleedings (TEER 4.6% vs. AP 2.7%) and no fatal bleedings occurred. Bleedings requiring intervention and red blood cell transfusions were significantly increased in the AP group.

Bleeding location in the TEER group was the upper gastrointestinal tract in two patients, the venous access site and the urinary bladder in one patient each and unknown in three patients. In the AP group there were predominantly access site bleedings with five venous and one arterial vascular access bleeding and upper gastrointestinal bleedings in five patients (of which two were bleedings of gastric ulcers or dysplasia and three were in the esophagus). There were overall four pericardial effusions in AP group treated with cardiothoracic surgery, pericardiocentesis, RCA stenting and conservatively in one patient each. The other bleeding locations were pretreated teeth and hemorrhoids, while one bleeding location was not localizable.

Rate of tricuspid valve reinterventions was higher in the TEER compared to the AP group, which was of borderline significance (8.0 vs. 1.4%, *p* = 0.061). Three of these patients in the TEER group underwent percutaneous tricuspid valve repair and four patients underwent surgical valve replacement, three of whom died. One patient in the AP group underwent TEER after unsuccessful annuloplasty in the same procedure.

### Clinical outcome of total cohort

**Fig. 3:**
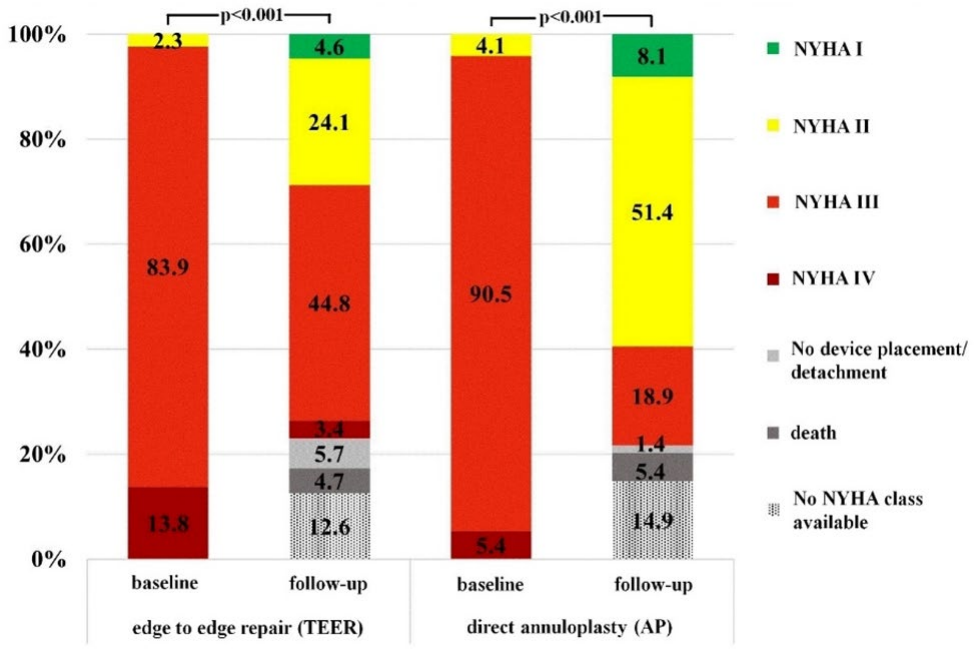
Comparison of NYHA functional class. For clinical outcome NYHA functional class of patients treated with transcatheter edge to edge repair and direct annuloplasty was compared at baseline and follow-up. Both treatment techniques significantly improved NYHA class, with a significantly more pronounced benefit in the annuloplasty group

NYHA class was assessed at the first outpatient appointment which was on average 95 days (± 77 days) after the procedure. NYHA class significantly improved in both groups with NYHA class I/II in 28.7% in the TEER group and in 59.5% in the AP group. Significantly more patients in the AP group showed a symptomatic benefit with improvement in NYHA class (Table 3; Fig. 3).

**Fig. 4:**
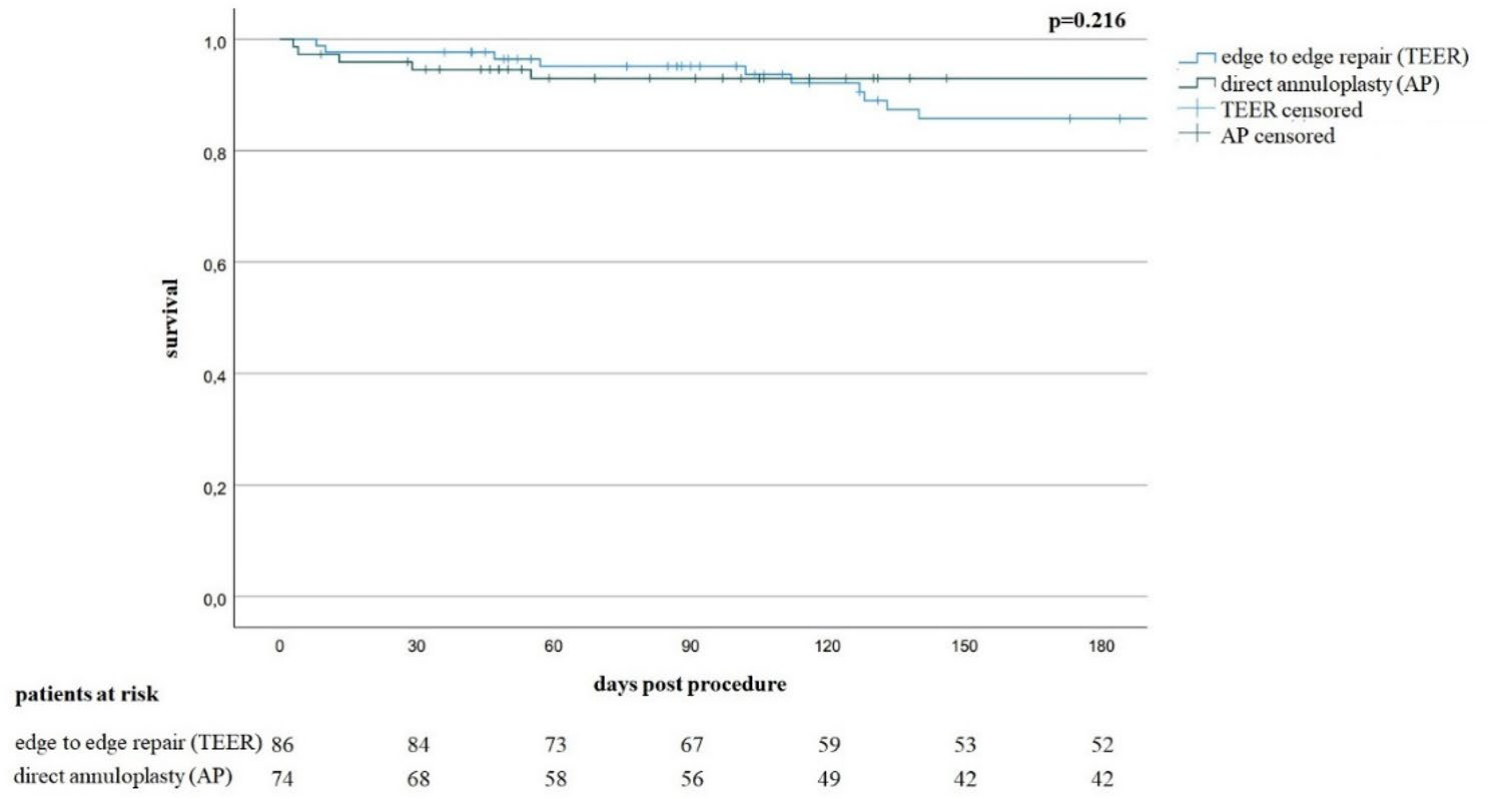
Six months survival by treatment group. For mid-term outcome the survival of patients treated with transcatheter edge to edge repair and direct annuloplasty was examined with Kaplan–Meier analysis. There is no significant difference in survival comparing both treatment groups (*p* = 0.216)

In addition to the death events during the initial hospital stay or 30-day follow-up, within 6-month follow-up 10 patients died in the TEER group and five patients died in the AP group. Total 6-month mortality was not significantly different across groups (Fig. 4).

## Discussion

To the best of our knowledge this is the first study to compare the most commonly used transcatheter repair techniques which emerged as important treatment options in patients with secondary TR and high surgical risk [18]. Patients of two high-volume centers treated with direct AP had overall comparable RV dimensions as patients with TEER therapy, but greater coaptation gaps and vena contracta widths with significantly higher TR grade at baseline. Comparable TR grades were achieved post-procedurally and analysis matched for differences in baseline TR severity demonstrated a significantly higher rate of optimal results with TR less than moderate in the AP group. Contrarily, longer procedural time and more bleeding complications were observed in the AP group.

The magnitude of baseline TR severity, procedural efficacy and safety of our cohort is comparable to results of the pivotal approval trials of the involved devices. Baseline frequency of torrential TR ranged from 37 to 56%, reduction to moderate TR or less ranged from 44 to 76%, and major bleeding events ranged from 6 to 23%, suggesting reasonable representativeness of the performance of our two centers [5, 10, 19].

So far, head-to-head comparisons of TEER and AP procedures are lacking. AP using Cardioband is more complex than TEER with respect to both patient screening which includes cardiac computed tomography and the procedure itself which results in substantially longer procedural duration. Although our two centers are very experienced in the AP procedure, duration still is by about 85 min longer than respective TEER procedures. Hence, in clinical practice usually TEER is the first treatment choice and the main reason to decide for AP is that TEER will not achieve a satisfying result based on the patient’s valve morphology. TEER therapy is limited when corresponding leaflets cannot be adequately grasped with the device, which occurs mainly when the gap between leaflets is too large, leaflet tethering is too pronounced or both in combination. Accordingly, in our cohort TR severity and associated parameters such as coaptation gap were significantly larger in the AP compared to the TEER group. Another small single-center study of patients undergoing Cardioband procedure also reported a higher rate of patients with torrential TR than in a contemporary large multicenter register of patients undergoing PASCAL implantation [20, 21].

Despite advanced baseline TR in the AP group, the postprocedural results were similar between AP and TEER treatments. This is of clinical relevance, since albeit much effort is put on promoting early detection and therapy of TR, currently a substantial part of patients with relevant TR is introduced at quite advanced stages [22]. Indeed, in TEER a large coaptation gap which is usually associated with advanced TR stages is an important risk factor of treatment failure and a coaptation gap of ≤ 8.4 mm has recently been proposed for optimal results in TEER [23].

When correcting for differences in baseline TR severity between groups, an optimal result with TR less than moderate was achieved more frequently with AP, whereas an acceptable result with TR less than severe was similar between groups. It is unclear whether the difference between moderate and mild residual TR will have clinical impact for the patients. Nonetheless, it seems comprehensible that a valvular pathology which is caused by annular dilatation can most effectively be treated with an AP technique. Leaflet morphology is quite complex in many patients with TR with more than three leaflets in about 40% of patients [24]. Usually TEER therapy addresses two commissures which necessarily means that one or two commissures are left untreated. Furthermore, the reductive effect on annular diameter is less in TEER than in AP therapy which finally might explain an increased rate of moderate residual TR in TEER [5].

MAE in first 30 days were similar in both groups. However, when further considering additional softer endpoints such as bleeding, access-site complications and renal failure overall complication rate was higher in the AP group. In literature this trend is underlined by 30-day MAE incidences in patients treated with TEER varying between 1.7 and 8.0% and in patients treated with AP between 13.3 and 19.7% [25–28]. In our analysis bleeding complications were more than twice as frequent after AP treatment as after TEER, which also might affect incidence of adverse events such as renal failure and reasons might be multifactorial. The additional arterial access necessary for coronary angiography in AP is a possible source for bleeding complications, albeit access site-related bleeding complication occurred predominantly on the venous side. Bleedings requiring interventions were mainly due to gastrointestinal bleeding with hemoptysis after transesophageal echocardiography. It has been shown earlier, that bleeding complications in percutaneous mitral valve repair are associated with longer procedures [19]. Hence, the significantly longer procedure time for AP is per se a risk factor for more bleeding complications. The complex echocardiography guiding in AP requires frequent changes between esophageal and transgastric views which is an additional trigger of gastrointestinal mucosa irritation and bleeding events. This tendency of more bleeding complications in AP treatment is congruent to previous studies with bleeding events in 13% in TRI-REPAIR (TrIcuspid Regurgitation RePAIr With CaRdioband Transcatheter System) study [10]. Comparing 1-year outcome data of feasibility studies with major bleeding events in 11.9% of patients treated with TEER and in 35.1% of patients treated with Cardioband a possible higher bleeding rate has to be considered for treatment decision in patients with high bleeding risk [7, 29]. Importantly, in our analysis live threatening bleedings were comparable between groups and fatal bleedings events did not occur. In comparison with recent studies as TRILUMINATE Pivotal Trial (The Trial to Evaluate Cardiovascular Outcomes in Patients Treated with the Tricuspid Valve Repair System Pivotal) incidences of adverse events appear higher in our real-world cohort [28]. This is most likely due to strict exclusion criteria in pivotal trials with overall healthier patient cohorts in contrast to our real-world patients as indicated, for example, by more advanced NYHA status, higher NT-pro-BNP levels and lower LV-EF.

Clinical benefit concerning NYHA functional class was more pronounced in the AP group. This might be explained by several differences in baseline characteristics between groups such as age, coronary heart disease, and right heart function, and also by the different contribution of TR to the overall symptom burden. Interestingly, a comparison of AP and TEER therapy in patients with secondary mitral regurgitation also showed a better clinical outcome and benefit in the AP compared to the TEER group which might warrant future comparison of both techniques in larger patient samples [30].

### Limitations

Several limitations must be considered. Inherent to the retrospective and observational study design are limitations such as incompleteness of data and selection bias with respect to the decided treatment technique. Not all patients underwent evaluation for AP and the major reason for selecting AP was potentially adverse morphology for TEER. We addressed the latter by performing a full matching analysis for the primary efficacy endpoint. An advantage of enrolling both treatment groups from the same centers is to avoid a systematic bias with respect to assessments of patient characteristics, endpoints and extra-procedural aspects of the intervention. Furthermore, both centers share a similar stage of experience for both techniques. An important limitation is the cohort size which limits statistical power to detect minor differences in efficacy and safety. Finally, we lumped together three different leaflet-based devices to the TEER group. We acknowledge that these devices differ in technical details which might translate in differences in efficacy and safety. Nonetheless, a recent study comparing the PASCAL system with Triclip did not show a significant superiority of either system [31].

## Conclusion

This analysis of contemporary patients undergoing either of the two most commonly used transcatheter repair techniques in TR demonstrated that despite having more severe TR grade at baseline AP showed a similar technical success and procedural efficacy in reducing TR. Propensity score matched analysis showed higher efficacy of AP with respect to optimal results with TR of less than moderate. However, AP takes significant more time and was associated with more non-fatal bleeding events. These first comparative findings provide evidence for tailoring distinct transcatheter treatment techniques to individual patient characteristics.

## Data Availability

The data that support the findings of this study are available from the corresponding author, [LO], upon reasonable request.

## Abbreviations

AP: Direct annuloplasty
BMI: Body mass index
CG: Coaptation gap
EROA: Effective regurgitant orifice area
TEER: Transcatheter edge-to-edge repair
MAE: Major adverse events
MVR: Mitral valve replacement
MVARC: Mitral Valve Academic Research Consortium
PMVR: Percutaneous mitral valve repair
PTVR: Percutaneous tricuspid valve repair
RCA: Right coronary artery
SPAP: Systolic pulmonary artery pressure
TAPSE: Tricuspid annular plane systolic excursion
TR: Tricuspid regurgitation
VC: Vena contracta
